# Supplementary material for: Predicting visual function by interpreting a neuronal wiring diagram
Source: Nature. 2024 Oct 2;634(8032):113–23. doi: 10.1038/s41586-024-07953-5 (PMC11446822; doi:10.1038/s41586-024-07953-5)
Supplement: Supplementary file 6 — CRF and ERF predictions for individual TmY4 and TmY9 cells. Analogous to Supplementary Data 3, but for TmY target types. Shown are the top four monosynaptic pathways, the strongest pathway passing through each of the top ten intermediary types (ranking from Extended Data Fig. 7), and the trisynaptic pathway Tm1–TmY–Dm3–TmY (see the section entitled Prediction of spatial normalization). [file 41586_2024_7953_MOESM6_ESM.zip › DataS4/TmY9q/720575940647073412.pdf]

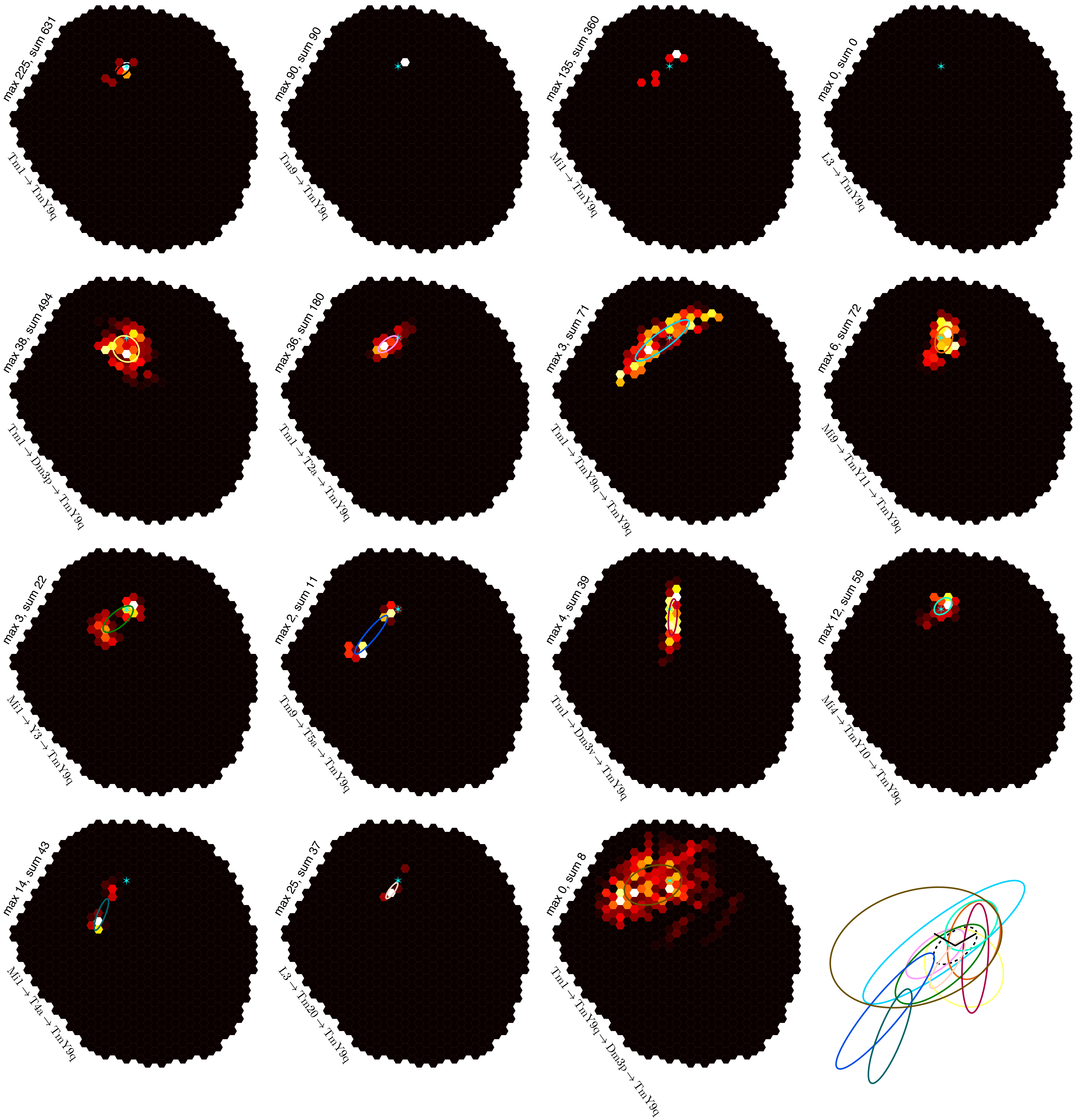
max 225, sum 631
Tm1 → TmY9q
max 90, sum 90
Tm9 → TmY9q
max 135, sum 360
Mi1 → TmY9q
max 0, sum 0
L3 → TmY9q
max 38, sum 494
Tm1 → Dm3p → TmY9q
max 36, sum 180
Tm1 → T2a → TmY9q
max 3, sum 71
Tm1 → TmY9q → TmY9q
max 6, sum 72
Mi9 → TmY11 → TmY9q
max 3, sum 22
Mi1 → Y3 → TmY9q
max 2, sum 11
Tm9 → T5a → TmY9q
max 4, sum 39
Tm1 → Dm3v → TmY9q
max 12, sum 59
Mi4 → TmY10 → TmY9q
max 14, sum 43
Mi1 → T4a → TmY9q
max 25, sum 37
L3 → Tm20 → TmY9q
max 0, sum 8
Tm1 → TmY9q → Dm3p → TmY9q
